# Physics-Guided Multi-Task Learning for Small-Sample Soft Sensing: Simultaneous Prediction of Kappa Number and Viscosity in Continuous Kraft Pulping

**DOI:** 10.3390/s26082395

**Published:** 2026-04-14

**Authors:** Bing Zhang, Liuxin Shi, Xiao Zhang, Minghui Xu

**Affiliations:** College of Light Industry and Food Engineering, Nanjing Forestry University, Nanjing 210037, China; bingzhang@njfu.edu.cn (B.Z.); 3231500679@njfu.edu.cn (X.Z.); 3168876459@njfu.edu.cn (M.X.)

**Keywords:** soft sensor, physics-guided learning, multi-task learning, small-sample modeling, Kappa number, pulp viscosity, continuous kraft pulping

## Abstract

In continuous kraft pulping, key quality indicators such as Kappa number and pulp viscosity are usually measured offline at low frequency, which limits real-time quality monitoring and control. Although data-driven soft sensors have shown potential for quality prediction, their performance is often restricted by limited labeled data and weak physical consistency. In addition, existing studies have focused mainly on single-target prediction, while the coupled prediction of Kappa number and pulp viscosity remains insufficiently explored despite their common dependence on cooking conditions and degradation kinetics. To address these issues, this study proposes a physics-guided multi-task learning framework (PG-MTL) for simultaneous prediction of Kappa number and pulp viscosity. The model combines a hard-parameter-sharing multi-task architecture with a physics-guided monotonicity constraint that enforces the expected non-increasing Kappa trend with increasing H-factor. Homoscedastic uncertainty weighting is also used to balance the two regression tasks during optimization. Experiments on industrial operating data show that PG-MTL achieved $R^{2}\;$= 0.920 for the Kappa number and $R^{2}$ = 0.910 for pulp viscosity. Compared with the strongest benchmark model, RMSE was reduced by 23.2% and 29.5% for the Kappa number and pulp viscosity, respectively. These results demonstrate that PG-MTL provides an effective and physically consistent solution for pulp-quality soft sensing under small-sample industrial conditions.

## 1. Introduction

The continuous kraft pulping process remains a cornerstone of modern pulp and paper manufacturing, functioning as a large-scale thermo-chemical reactor in which lignin is selectively removed from wood chips to liberate high-quality cellulosic fibers [1]. Effective process control demands a delicate balance between delignification and carbohydrate preservation [2,3]. Pulp quality is routinely assessed by two key indicators: the Kappa number, an indirect measure of residual lignin content, and pulp viscosity, which reflects the degree of polymerization of cellulose and hence intrinsic fiber strength. In industrial practice, however, both variables are determined through offline laboratory analyses performed at intervals of several hours. The resulting feedback delay severely limits real-time optimization and motivates the development of reliable soft sensors capable of predicting quality trajectories from readily available online process variables [4]. Consequently, the development of robust, real-time soft sensors, particularly under conditions of limited labeled data in industrial processes capable of predicting quality trajectories based on online process variables, has become an imperative research direction.

Soft-sensing strategies in the pulp and paper industry have progressed from mechanism-based inferential estimation to data-driven prediction models [5,6]. In continuous kraft digesters, early efforts largely relied on first-principles or reduced-order models constructed from pulping kinetics, mass and energy balances, and simplified process dynamics to estimate hard-to-measure quality variables such as the Kappa number [7,8]. Although these models provide a physically interpretable description of the cooking process, their industrial applicability is often limited. The main challenges arise from the complexity of digester operation, long residence times, multirate measurements, and the difficulty of maintaining model accuracy under changing wood properties, liquor chemistry, and process disturbances [1,3]. Data-driven methods provide a more flexible alternative by learning input-output relationships directly from plant data. In existing pulp and paper studies, statistical and machine-learning models, such as multiple linear regression [9], ARIMA [10], artificial neural networks [11,12,13], and recurrent neural networks [12,14] have been used for Kappa number prediction [12,15,16], paper-property estimation, and equipment-condition forecasting [17,18,19]. These studies suggest that data-driven soft sensors are effective for describing nonlinear relationships in pulping processes. However, for industrial cooking applications, predictive accuracy alone is insufficient. The model should also remain robust and consistent with known delignification and cooking mechanisms [20,21,22].

In recent years, physics-guided or physics-informed learning has attracted increasing attention in industrial process monitoring and soft sensing because it can improve robustness and interpretability by embedding process knowledge into data-driven models. Recent studies have reported physics-guided graph learning soft sensors, physical-anchored graph learning frameworks, hybrid soft sensors that integrate process mechanisms with deep learning, and physics-informed industrial process models with actual plant validation [23,24,25,26]. However, these methods have been developed mainly for general chemical or process-industry applications, or for single-target prediction tasks, rather than for the coupled prediction of multiple quality indicators in continuous kraft pulping. Consequently, the specific combination of sparse labeled data, strong physicochemical coupling, and known cooking kinetics in continuous kraft pulping remains insufficiently addressed.

Nevertheless, the practical deployment of deep learning in the continuous kraft pulping industry faces two persistent challenges. First, labeled data are inherently scarce because Kappa number and pulp viscosity are obtained from infrequent laboratory assays rather than continuous online analyzers, and the number of usable industrial samples is further reduced after synchronization, cleaning, and steady-state screening. Training high-capacity neural networks on such limited data readily leads to overfitting, where models capture noise rather than underlying process kinetics [27]. Second, purely data-driven models do not guarantee consistency with known cooking behavior. In particular, the Kappa response should remain physically consistent with increasing H-factor under comparable chemical conditions. Although physics-informed learning has shown promise in broader industrial applications, its use in continuous kraft pulping remains limited, especially for jointly modeling coupled quality indicators under small-sample conditions [6,23,28,29].

An additional limitation in the existing literature is the common practice of treating Kappa number and pulp viscosity as independent prediction tasks [1,30]. Existing soft-sensing studies in kraft pulping have focused predominantly on single-target prediction, especially Kappa number, while the coupled prediction of Kappa number and pulp viscosity has received much less attention. In reality, however, these two indicators are intrinsically coupled through shared degradation kinetics governed by the same process variables, notably the H-factor, effective alkali concentration, and sulfidity [31,32]. Treating them separately therefore overlooks potentially useful shared information and may lead to less consistent predictions. Multi-task learning (MTL) provides a suitable framework for exploiting this coupling by learning a shared representation for related tasks [19,33], which is particularly valuable under noisy and limited-data industrial conditions. Existing MTL-based approaches in industrial soft sensing are usually designed to exploit statistical task relatedness, but they rarely incorporate process-specific physical priors to constrain the shared representation toward kinetically plausible solutions. Nevertheless, the incorporation of physics-guided constraints into such a multi-task framework remains insufficiently explored in continuous kraft pulping.

To address the above challenges, this study develops a physics-guided multi-task learning (PG-MTL) framework for robust soft sensing in continuous pulping. Most existing studies have focused on single-target prediction. However, the joint prediction of Kappa number and pulp viscosity remains insufficiently explored, even though both variables depend on the same cooking conditions and degradation kinetics. The proposed framework combines process knowledge with deep representation learning to improve prediction reliability under limited labeled data. A hard-parameter-sharing architecture is used to jointly model the Kappa number and pulp viscosity, enabling the network to capture their intrinsic coupling. In addition, a physics-guided loss term derived from H-factor kinetics is introduced to regularize the model according to the expected monotonic trend between cooking severity and residual lignin content. This improves physical plausibility and helps reduce overfitting. A homoscedastic uncertainty-based weighting strategy is further employed to adaptively balance the two tasks during training. Validation on industrial operating data shows that the PG-MTL model achieves better predictive accuracy and generalization than conventional single-task and purely data-driven benchmark models. The proposed approach therefore provides a practical and physically consistent solution for real-time pulp quality monitoring.

The remainder of this paper is organized as follows. Section 2 introduces the industrial continuous kraft pulping process and the dataset used in this study. It also analyzes the physical and statistical characteristics that motivate the subsequent model design. Section 3 presents the proposed physics-guided multi-task learning (PG-MTL) framework. Section 4 reports the experimental results and discussion, including predictive performance, robustness, and physical consistency. Section 5 concludes the paper and outlines future work.

## 2. Process Description and Data Analysis

### 2.1. Process Mechanism and Reaction Kinetics

**Figure 1:**
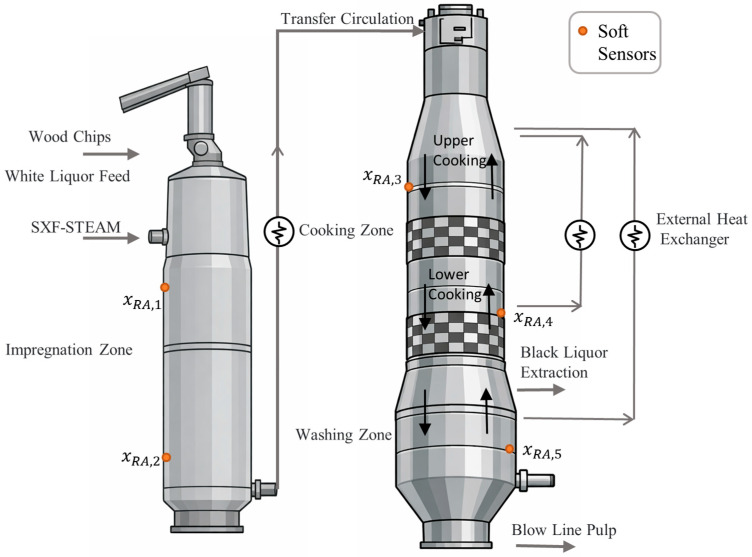
Schematic of the two-vessel continuous kraft pulp digester system.

The industrial process examined in this study is a commercial two-vessel hydraulic continuous kraft digester system [1,34], as shown schematically in Figure 1. Unlike conventional single-vessel designs, this configuration physically separates the impregnation stage from the main chemical reaction stage. This separation enhances liquor penetration and improves pulp uniformity [1]. Pre-steamed wood chips are first fed into the impregnation vessel (Figure 1, left tower), where they are contacted with white liquor, an aqueous solution of sodium hydroxide and sodium sulfide, at a moderate temperature. This stage mainly facilitates the diffusion of active alkali ions into the porous wood structure while limiting premature delignification [35]. After impregnation, the chip slurry is hydraulically transferred to the main vapor–liquid phase digester (Figure 1, right tower). It then descends through several functional zones, including heating, cooking, and counter-current washing sections. The system typically operates under a modified continuous cooking (MCC) strategy, in which white liquor is injected at multiple circulation points to maintain a relatively uniform effective alkali concentration along the digester height. Within the cooking zone, the temperature is increased to approximately 150–170 °C to initiate bulk delignification reactions. Under these conditions, lignin is progressively depolymerized and dissolved into the circulating black liquor [3], and the dissolved lignin fragments are then removed with the liquor stream. Although elevated temperature and alkali concentration accelerate lignin removal, the same thermo-chemical environment also promotes the random scission of cellulose chains through alkaline hydrolysis and peeling reactions. As a result, the cooking process must be carefully controlled to balance the reduction in residual lignin, quantified by the Kappa number, with the preservation of pulp viscosity. The latter reflects the degree of polymerization of cellulose and is closely related to fiber strength.

To quantitatively characterize the intensity of the thermo-chemical reactions occurring during kraft pulping, the process state is commonly described using Vroom’s H-factor [36]. This is a unified kinetic variable that integrates the temperature-dependent reaction rate over the residence time of the wood chips. Derived from the Arrhenius relationship, the H-factor represents the cumulative thermal severity experienced by the material along its trajectory through the digester and is expressed as [2]:

(1) $$H=\int_{0}^{t}k_{rel}(T)dt=\int_{0}^{t}\exp\left(43.2-\frac{16113}{T(t)}\right)dt$$

where *T*(*t*) is the absolute temperature (K) at time t, and the constants are derived from the activation energy of bulk delignification for typical softwood species. As an intermediate physical variable, the H-factor serves as a direct measure of cooking severity. It captures a fundamental trade-off in the pulping process: higher H-factor values promote lignin removal, but they also accelerate random alkaline hydrolysis and peeling reactions in cellulose chains, thereby reducing the degree of polymerization (DP) and pulp viscosity [32].

In industrial practice, this coupled reaction behavior is reflected in two key quality indices: the Kappa number, which indirectly measures residual lignin content, and pulp viscosity, which indicates the degree of polymerization of cellulose and is closely related to fiber strength. Under relatively stable alkali conditions, increasing cooking severity (that is, a higher H-factor) promotes lignin removal while simultaneously degrading carbohydrates. This fundamental trade-off can be expressed by the following monotonic physical constraint [3]:

(2) $$\frac{\partial y_{k}}{\partial x_{H}}<0$$

This relationship provides a useful physical prior for model development. When training data are limited, noise and sparse sampling may cause purely data-driven models to deviate from this constraint. By introducing it as a regularization term, the proposed model can better maintain consistency with the irreversible nature of delignification, while still describing the coupled evolution of Kappa number and pulp viscosity.

### 2.2. Data Description and Variable Selection

The industrial dataset used in this study was collected from a large-scale continuous kraft pulping line in Hainan Province, China, over a six-month production period from March to September 2024. This time span captures long-term process dynamics, including seasonal variation and changes in hardwood feedstock properties. Process variables were recorded at high frequency by the distributed control system (DCS). These data were then synchronized with low-frequency laboratory measurements of Kappa number ($y_{\kappa }$) and pulp viscosity ($y_{v}$), which were analyzed approximately every eight hours. In practical terms, this sampling interval yields only about three labeled quality samples per day, even though process conditions may vary continuously due to routine operating adjustments and unmeasured disturbances, such as variations in wood-chip moisture, chip composition, and liquor chemistry. Since the residence time of the digester is on the order of several hours, these low-frequency measurements are insufficient to resolve many intermediate variations and transient deviations that occur within the process. For each discharge sample, the input features were calculated as moving averages over the corresponding residence-time window. As a result, the present problem is not only small-sample in the conventional statistical sense, but is also characterized by a substantial observability gap between high-frequency process evolution and low-frequency quality labels.

To construct a compact but kinetically informative input space, eight variables were selected based on mechanistic relevance and measurement availability. The H-factor ($x_{H}$) was used as the primary thermodynamic descriptor because it integrates the entire temperature profile. As a result, individual zonal temperatures and raw residence times were treated as redundant [37]. Chemical driving forces were represented by effective alkali (EA) and sulfidity. In addition, residual alkali (RA) concentrations measured at five strategic locations ($x_{RA,1}$ to $x_{RA,5}$) were used to describe the spatial trajectory of alkali consumption. These RA measurements act as real-time proxies for unmeasured disturbances, such as variations in wood-chip digestibility. In the present study, the five RA measurements were treated as fixed-position process descriptors rather than as a long spatial sequence, because the limited number of spatial points and the relatively small dataset make more complex recurrent or convolution-based architectures less suitable for the current modeling scope. Although the MCC strategy involves split white-liquor addition and the digester consists of two vessels, a single overall H-factor was considered adequate for the present modeling scope. This is because it cumulatively reflects the thermal exposure over the full process trajectory [1,3].

**Figure 2:**
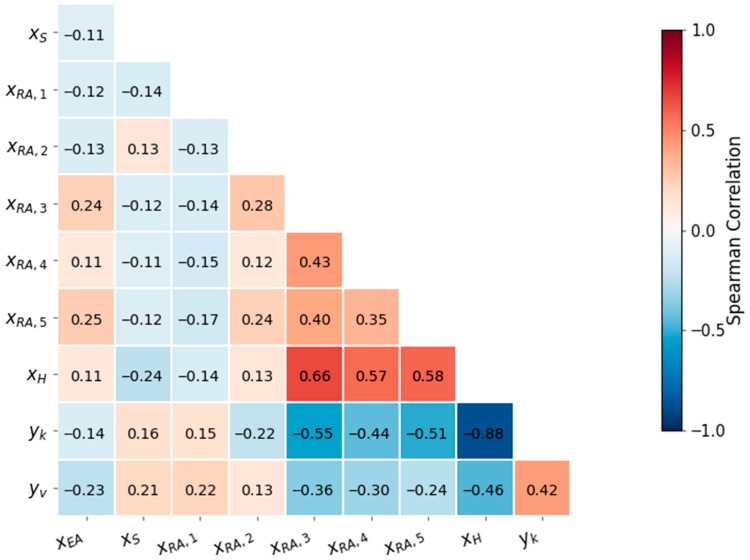
Spearman rank correlation heatmap showing the relationships between the selected process variables and the two pulp quality indicators (Kappa number $y_{k}$ and pulp viscosity $y_{v}$). All correlations are computed on the full industrial dataset after preprocessing.

Table 1 summarizes the selected input and output variables, including their symbols, physical meanings, units, and operating ranges. To examine the kinetic relevance of the selected variables, a nonparametric correlation analysis was performed, and the results are shown in Figure 2. The H-factor exhibited a strong negative correlation with the Kappa number (ρ = −0.88), confirming its dominant association with delignification severity, and a moderate negative correlation with pulp viscosity (ρ = −0.46), reflecting the concurrent degradation of carbohydrates under intensified cooking conditions. In addition, the Kappa number and pulp viscosity showed a moderate positive inter-correlation (Spearman ρ = 0.415, 95% CI [0.337, 0.484]; Pearson r = 0.391, 95% CI [0.316, 0.460]). This result suggests that the two quality indices are influenced by partially shared process conditions, while still retaining distinct task-specific characteristics. Therefore, rather than being treated as completely independent targets, they are more appropriately viewed as related response variables arising from the same thermo-chemical environment. This observation, together with their common dependence on cooking severity and alkali conditions, supports the use of a multi-task learning framework to capture shared process representations while preserving task-specific output behavior [32].

## 3. Methodology

### 3.1. Data Preprocessing

Raw data collected from the continuous kraft cooking process were systematically preprocessed to improve data quality and ensure compatibility with the predictive models. Industrial measurements often contain noise, transient fluctuations, and outliers caused by sensor faults or operating disturbances. Therefore, several preprocessing steps were applied before model development, including outlier removal, steady-state screening, and data normalization.

Abnormal observations were detected using the isolation forest (iForest) algorithm [38], which is well suited for industrial datasets because it isolates anomalies through recursive random partitioning without imposing strong distributional assumptions. Samples identified as outliers were removed before subsequent analysis. To further improve the consistency of the dataset, a steady-state detection (SSD) procedure [39,40] was then applied to extract representative steady operating samples. This step was motivated by the fact that transient conditions caused by grade changes, production adjustments, or load fluctuations may obscure the underlying relationship between process variables and final pulp quality. The retained samples therefore correspond to relatively stable operating periods that are more suitable for the present soft-sensing task. 

After outlier removal and steady-state screening, the dataset was divided into training and test subsets. All variables were then standardized using Z-score normalization, where the mean and standard deviation were computed from the training set only and subsequently applied to the test set. This transformation removes scale differences among variables and facilitates stable model training:

(3) $$x_{scale}=\frac{x-x_{mean}}{x_{std}}$$

where *x* denotes the original value, $x_{mean}$ and $x_{std}$ are the mean and standard deviation of the corresponding variable estimated from the training set, and $x_{scale}$ is the normalized value.

After preprocessing, 478 valid samples were retained. To preserve the temporal structure of the industrial process data and to avoid information leakage between past and future observations, the dataset was divided in chronological order. The first 382 samples were used for model training, and the remaining 96 samples were reserved as an independent test set for evaluating generalization performance. Because the dataset spans a six-month industrial production period, some degree of process drift cannot be excluded. The chronological split therefore reflects not only interpolation accuracy but also the model’s ability to generalize across temporally separated operating data. Although the major process variables did not show an obvious descriptive mismatch between the training and test windows, the test set should still be interpreted as a temporally shifted operating window rather than an identically distributed sample.

### 3.2. Physics-Guided Multi-Task Learning (PG-MTL) Architecture

**Figure 3:**
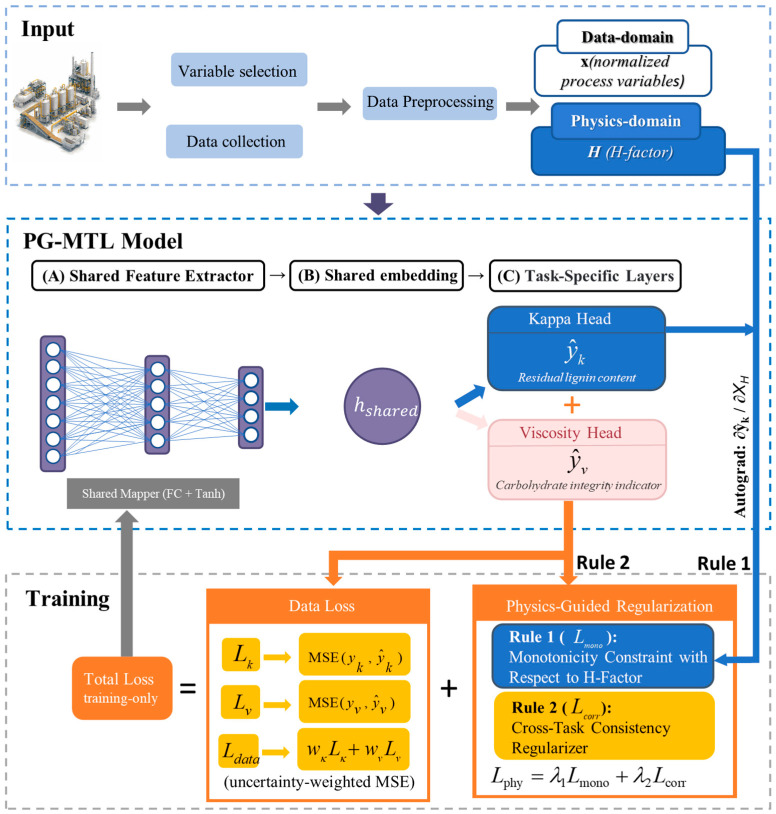
Schematic illustration of the proposed PG-MTL framework for simultaneous prediction of the Kappa number and pulp viscosity.

To predict the Kappa number and pulp viscosity under limited-data industrial conditions, a physics-guided multi-task learning (PG-MTL) framework was developed. As illustrated in Figure 3, the framework consists of three parts: an input module for variable construction and preprocessing, a shared-and-branch neural architecture for dual-target prediction, and a training objective that combines task losses with process-informed regularization. The model uses a shared feature extractor to learn common process representations, followed by two task-specific heads for Kappa number and viscosity prediction, respectively.

#### 3.2.1. Shared Feature Representation

The shared feature extractor is implemented as a compact multilayer perceptron (MLP) that maps the input variables into a latent representation of the process state. This module is intended to capture process information that is relevant to both delignification and carbohydrate degradation. Considering the limited size of the industrial dataset, a shallow shared trunk was adopted to balance expressive capacity and overfitting risk. Specifically, the shared network contains two fully connected layers with 64 and 32 neurons, respectively, and uses the hyperbolic tangent (Tanh) activation function in both hidden layers.

Given an input vector *x*, the shared latent representation *h* is expressed as:

(4) $$h=f_{shared}(x)$$

where $f_{shared}$(·) denotes the non-linear mapping learned by the shared network. The use of a shared latent representation is motivated by the fact that the Kappa number and pulp viscosity are influenced by the same thermo-chemical environment and therefore depend on partially overlapping process information. Hard parameter sharing in the lower layers enables the model to use this common information more efficiently under small-sample conditions.

#### 3.2.2. Task-Specific Prediction Heads

The shared representation *h* is then fed into two task-specific branches for predicting the Kappa number and pulp viscosity, respectively. Each branch contains one fully connected hidden layer with 16 neurons, followed by a linear output layer. The two outputs are written as:

(5) $$\left\{\begin{array}{l} \begin{array}{l} {\hat{y}}_{k}=f_{k}(h)\\ {\hat{y}}_{v}=f_{v}(h) \end{array} \end{array}\right.$$

where $f_{k}$(·) and $f_{v}$(·) denote the nonlinear mappings of the Kappa and viscosity heads, respectively. This architecture allows the model to preserve shared process information in the lower layers while retaining sufficient flexibility to capture target-specific response characteristics in the upper layers.

#### 3.2.3. Physics-Guided Regularization

To improve physical consistency under limited-data conditions, physics-guided regularization was incorporated into model training using additional constraint terms derived from the process knowledge summarized in Section 2. Unlike classical PINN formulations that typically embed governing differential equations or hard physical constraints directly into the training process, the proposed PG-MTL framework adopts a penalty-based regularization strategy. In the present study, process knowledge is introduced as soft constraints at the loss level, which guides optimization toward physically plausible solutions without imposing explicit equation-based restrictions. This design is more suitable for the current industrial setting, where an explicit and sufficiently accurate first-principles dynamic formulation for continuous kraft pulping is difficult to specify under realistic operating conditions. In the proposed PG-MTL framework, two regularization terms were considered. The primary physics-guided term is a monotonicity constraint imposed on the predicted Kappa number with respect to the H-factor, reflecting the expected delignification trend under increasing cooking severity. A cross-task consistency regularizer was further introduced to preserve the empirical coupling between the Kappa number and pulp viscosity during joint learning.

Rule 1: Monotonicity Constraint with Respect to H-Factor. 

As discussed in Section 2, increasing cooking severity is generally associated with continued delignification under comparable chemical conditions. The monotonicity constraint was imposed only with respect to the H-factor because, among the selected variables, the H-factor provides the most direct and robust descriptor of cumulative cooking severity. Under comparable chemical conditions, its relationship with delignification is more direct and therefore more suitable for monotonic regularization. By contrast, the effects of effective alkali, sulfidity, and residual alkali distribution on the Kappa response are more strongly coupled with local chemical conditions, liquor distribution, and process interactions, making it difficult to formulate a single monotonic prior for these variables without introducing overly restrictive assumptions. These variables were therefore retained as data-driven inputs, while the H-factor was selected as the primary carrier of process-based monotonic information. To encode this prior into the model, a gradient-based monotonicity constraint was imposed on the predicted Kappa number with respect to the H-factor input:

(6) $$\frac{\partial {\hat{y}}_{k}}{\partial x_{H}}<0$$

where $x_{H}$ denotes the H-factor input variable and ${\hat{y}}_{k}$ is the predicted Kappa number. During training, this partial derivative was computed by automatic differentiation with respect to the normalized input. Since Z-score normalization preserves the ordering of H-factor values, the sign of the monotonicity constraint remains unchanged in the transformed input space.

To penalize violations of the expected monotonic trend, a ReLU-based loss term was introduced:

(7) $$L_{\mathrm{mono}}=\frac{1}{N}\sum_{i=1}^{N}\mathrm{ReLU}\left(\frac{\partial {\hat{y}}_{k,i}}{\partial x_{H,i}}\right)$$

This penalty is activated only when the predicted gradient becomes positive, thereby discouraging trend reversals that are inconsistent with the expected delignification behavior while avoiding the rigidity of a hard constraint. The monotonicity penalty was computed directly by automatic differentiation with respect to the normalized H-factor input. No additional finite-difference smoothing or separate gradient-clipping strategy was applied specifically to this term. In practice, the use of smooth Tanh activations and mini-batch optimization provided sufficiently stable gradient estimates for training under the present setting.

Rule 2: Cross-Task Consistency Regularizer. 

In addition to the physics-guided monotonicity prior, a cross-task consistency regularizer was introduced to reflect the empirical interdependence between the Kappa number and pulp viscosity observed in the industrial dataset. As shown in Section 2, the two quality indicators exhibit moderate positive co-variation under the operating conditions considered here. To reduce the risk that the two task-specific branches evolve toward mutually inconsistent prediction patterns, a batch-wise correlation regularizer was defined.

For a mini-batch of predictions ${\hat{y}}_{k}={\left[{\hat{y}}_{k,1},\ldots ,{\hat{y}}_{k,B}\right]}^{T}$ and ${\hat{y}}_{v}={\left[{\hat{y}}_{v,1},\ldots ,{\hat{y}}_{v,B}\right]}^{T}$, the predicted Pearson correlation is computed as:

(8) $$\rho ({\hat{y}}_{k},{\hat{y}}_{v})=\frac{\sum_{i=1}^{B}\left({\hat{y}}_{k,i}-{\bar{\hat{y}}}_{k})({\hat{y}}_{v,i}-{\bar{\hat{y}}}_{v}\right)}{\sqrt{\sum_{i=1}^{B}{\left({\hat{y}}_{k,i}-{\bar{\hat{y}}}_{k}\right)}^{2}}\sqrt{\sum_{i=1}^{B}{\left({\hat{y}}_{v,i}-{\bar{\hat{y}}}_{v}\right)}^{2}}+\delta }$$

where ${\bar{\hat{y}}}_{k}$ and ${\bar{\hat{y}}}_{v}$ denote the batch means, and *δ* is a small positive constant introduced for numerical stability. To prevent the predicted coupling from falling below a plausible lower bound, a hinge-style penalty was used:

(9) $$L_{corr}=max(0,\rho_{\min}-\rho ({\hat{y}}_{k},{\hat{y}}_{v}))$$

where $\rho_{min}$ is specified according to the empirical correlation observed in the training set. This term does not impose a fixed linear relationship between the two targets; rather, it acts as a weak regularizer to preserve their minimum empirical coupling during optimization. 

The overall physics-guided regularization term is defined as:

(10) $$L_{\mathrm{phy}}=\lambda_{1}L_{mono}+\lambda_{2}L_{corr}$$

where $\lambda_{1}$ and $\lambda_{2}$ control the strengths of the monotonicity and cross-task consistency terms, respectively. This regularization term is jointly optimized with the uncertainty-weighted multi-task data loss described in Section 3.2.4.

#### 3.2.4. Uncertainty-Weighted Multi-Task Optimization

Because the Kappa number and pulp viscosity differ in scale, unit, and noise characteristics, assigning fixed loss weights manually may bias training toward one task. To address this issue, homoscedastic uncertainty weighting was adopted to adaptively balance the two regression objectives during optimization [41].

As illustrated in Figure 3, the data-driven component of the PG-MTL framework is formulated as a weighted combination of the two task losses. Let $L_{k}$ and $L_{v}$ denote the prediction losses for the Kappa number and pulp viscosity, respectively. In this study, both tasks were formulated as regression problems using mean squared error (MSE):

(11) $$L_{k}=MSE\left(y_{k},{\hat{y}}_{k}\right),L_{v}=MSE(y_{v},{\hat{y}}_{v})$$

The weighted data loss can then be written as:

(12) $$L_{data}=w_{k}L_{k}+w_{v}L_{v}$$

where $w_{k}$ and $w_{v}$ denote the task-specific weighting coefficients. Instead of assigning these weights manually, they are derived from task-dependent homoscedastic uncertainty. Assuming Gaussian observation noise for the two regression tasks with variances $\sigma_{k}^{2}$ and $\sigma_{v}^{2}$, the joint negative log-likelihood can be written as:

(13) $$L_{data}=\frac{1}{2\sigma_{k}^{2}}L_{k}+\frac{1}{2\sigma_{v}^{2}}L_{v}+\log\sigma_{k}+\log\sigma_{v}$$

Under this formulation, the effective task weights are learned automatically during training, with $w_{k}=\frac{1}{\sigma_{k}^{2}}$ and $w_{v}=\frac{1}{\sigma_{v}^{2}}$. In implementation, the corresponding log-variance terms $s_{k}=\log\left(\sigma_{k}^{2}\right)$ and $s_{v}=\log\left(\sigma_{v}^{2}\right)$ are optimized jointly with the network parameters. As a result, tasks associated with higher uncertainty receive smaller effective weights, whereas tasks with lower uncertainty contribute more strongly to parameter updates. This adaptive strategy improves optimization stability and reduces the risk that one task dominates the shared representation learning process. Here, the Gaussian assumption is used primarily to derive the homoscedastic uncertainty-weighted loss, rather than as a strict claim that the industrial measurement errors exactly follow Gaussian distributions. The uncertainty formulation should therefore be interpreted mainly as an adaptive task-balancing mechanism rather than a complete probabilistic error model.

The uncertainty-weighted data loss is integrated with the physics-guided constraints introduced in Section 3.2.3. The final objective function of the PG-MTL model is expressed as:

(14) $$L_{total}=L_{data}+\lambda_{1}L_{mono}+\lambda_{2}L_{corr}$$

where $L_{mono}$ and $L_{corr}$ denote the monotonicity and correlation consistency penalties, respectively, and $\lambda_{1}$ and $\lambda_{2}$ control the strength of the physics-guided constraints. This formulation enables the model to jointly optimize prediction accuracy, cross-task consistency, and physical plausibility within a unified training framework.

### 3.3. Experimental Setup and Implementation Details

To evaluate the proposed PG-MTL framework, comparative experiments were conducted using four representative baseline models: Partial Least Squares (PLS), Support Vector Regression (SVR) with a radial basis function (RBF) kernel, Extreme Gradient Boosting (XGBoost), and a vanilla multi-task deep neural network (MTL-DNN). PLS was included as a classical linear baseline for multicollinear industrial data. SVR and XGBoost were used as representative nonlinear machine-learning models, while MTL-DNN served as a purely data-driven deep-learning baseline. The MTL-DNN model used the same shared-and-branch architecture as PG-MTL but was trained without the physics-guided regularization and uncertainty-weighted learning mechanism, thereby functioning as a purely data-driven reference model. Both deep-learning models were developed under the assumption that all eight selected input variables were available after preprocessing. Accordingly, the present architecture does not explicitly support partially missing inputs at inference time and does not include an internal imputation mechanism; in practical deployment, such cases would need to be handled upstream through data preprocessing or imputation before the complete feature vector is passed to the model. For SVR and XGBoost, hyperparameters were optimized by grid search with 10-fold cross-validation on the training set only. Bayesian optimization was not employed for the deep-learning models. Instead, a systematic hyperparameter screening was conducted on the validation set to establish a robust architecture for the purely data-driven MTL-DNN. The same architectural and training configuration was then used for PG-MTL, so that the effect of physics-guided regularization could be assessed under the same backbone and optimization protocol. This controlled design was adopted to reduce confounding from separately optimized search budgets and to preserve a fair structural comparison between the two deep-learning models. All model selection procedures were therefore performed without using the independent test set. The predictive performance of all candidate models was evaluated on the independent test set using three standard regression metrics: Root Mean Square Error (RMSE), Mean Absolute Error (MAE), and the coefficient of determination ($R^{2}$). These metrics are defined as follows:

(15) $$RMSE=\frac{1}{N_{t}}\sqrt{\sum_{i=1}^{N_{t}}{\left({\hat{y}}_{i}-y_{i}\right)}^{2}}$$

(16) $$MAE=\frac{1}{N_{t}}\sum_{i=1}^{N_{t}}|{\hat{y}}_{i}-y_{i}|$$

(17) $$R^{2}=1-\frac{\sum_{i=1}^{N_{t}}{\left(y_{i}-{\hat{y}}_{i}\right)}^{2}}{\sum_{i=1}^{N_{t}}{\left(y_{i}-\bar{y}\right)}^{2}}$$

where *N* is the sample size, ${\hat{y}}_{i}$ and $y_{i}$ denote the predicted and measured values, respectively, and $\bar{y}$ is the mean of the measured outputs.

The deep-learning models were implemented in PyTorch (version 2.4.0) under Python 3.12, while the conventional baseline models were implemented using Scikit-learn. The PG-MTL network used a shared feature extractor with two hidden layers of 64 and 32 neurons, followed by two task-specific heads, each containing one hidden layer of 16 neurons. Tanh activation was used in all hidden layers because the monotonicity regularization requires smooth and differentiable gradients with respect to the H-factor input. Model parameters were optimized using Adam with an initial learning rate of 0.001. A ReduceLROnPlateau scheduler was used to decrease the learning rate by a factor of 0.5 when the validation loss failed to improve for 10 epochs. In the present study, the scheduler and early stopping were used jointly for training stabilization. In practice, the validation loss typically entered a plateau region before the maximum epoch limit was reached, after which the scheduler reduced the learning rate, and training continued until the early-stopping criterion was met if no further improvement was observed. Although early stopping can help limit overfitting, it does not replace the structural regularization effect introduced by the physics-guided constraint under limited-data conditions. To reduce overfitting, 15% of the training data were further held out as a validation subset. The batch size was set to 32, the maximum number of training epochs was 500, and early stopping was triggered if the validation loss did not improve for 30 consecutive epochs.

The regularization hyperparameters were selected based on validation performance within the training data. Specifically, the monotonicity penalty weight $\lambda_{1}$ and the cross-task consistency weight $\lambda_{2}$ were tuned to balance predictive accuracy against the strengths of the two regularization terms. Values that were too small weakened the intended regularization effect, whereas overly large values impaired data fitting. Based on this trade-off, $\lambda_{1}$ and $\lambda_{2}$ were set to 0.1 and 0.05, respectively. The lower-bound correlation threshold $\rho_{min}$ was set to 0.35 according to the empirical Pearson correlation observed in the training set, so that the consistency term preserves a minimum level of coupling between the two targets without enforcing a fixed linear relationship. In addition, the uncertainty-weighted multi-task optimization follows the homoscedastic uncertainty formulation proposed by Kendall et al. [41]. To improve numerical stability, the task uncertainties were parameterized in log-variance form, i.e., $s_{k}=\log\left(\sigma_{k}^{2}\right)$ and $s_{v}=\log\left(\sigma_{v}^{2}\right)$. The corresponding log-variance terms were treated as trainable parameters rather than fixed hyperparameters. They were initialized at zero and optimized jointly with the network weights using the same optimizer and learning-rate schedule, allowing the model to adaptively learn task-specific noise levels while ensuring positive effective variances throughout training. The complete hyperparameter settings are summarized in Table 2.

All computational experiments were conducted on a workstation equipped with an Intel^®^ Core™ i9-13900HX CPU and an NVIDIA GeForce RTX 4080 GPU. To improve reproducibility, a fixed random seed of 42 was used in all experiments.

## 4. Results and Discussion

This section evaluates the proposed PG-MTL framework from four complementary perspectives. First, overall predictive performance is assessed by comparing PG-MTL with representative linear, kernel-based, ensemble, and deep-learning benchmarks using error metrics and structural statistical analyses. Second, an ablation study is conducted to examine the respective contributions of multi-task learning and physics-guided regularization. Third, robustness under reduced training data is investigated to assess performance stability in small-sample settings. Finally, physical consistency is examined through an H-factor stress test to determine whether the learned mapping remains consistent with the expected cooking kinetics.

### 4.1. Overall Prediction Performance

**Figure 4:**
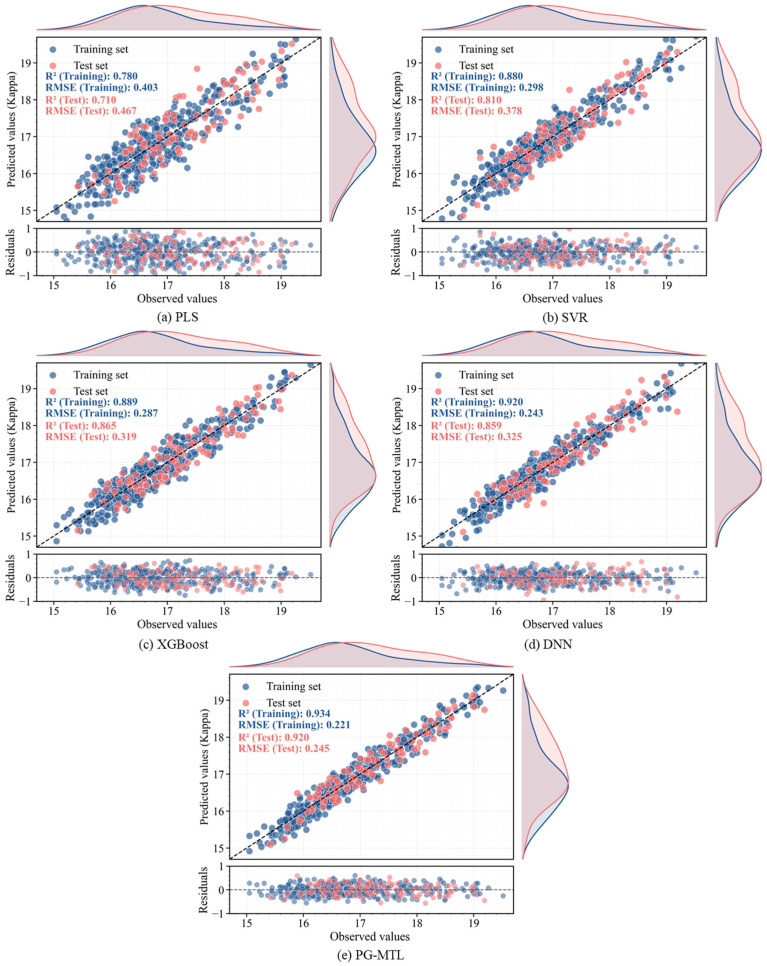
Parity and residual plots for Kappa number prediction: (**a**) PLS, (**b**) SVR, (**c**) XGBoost, (**d**) MTL-DNN, and (**e**) proposed PG-MTL. Training and test sets are shown in blue and red, respectively.

**Figure 5:**
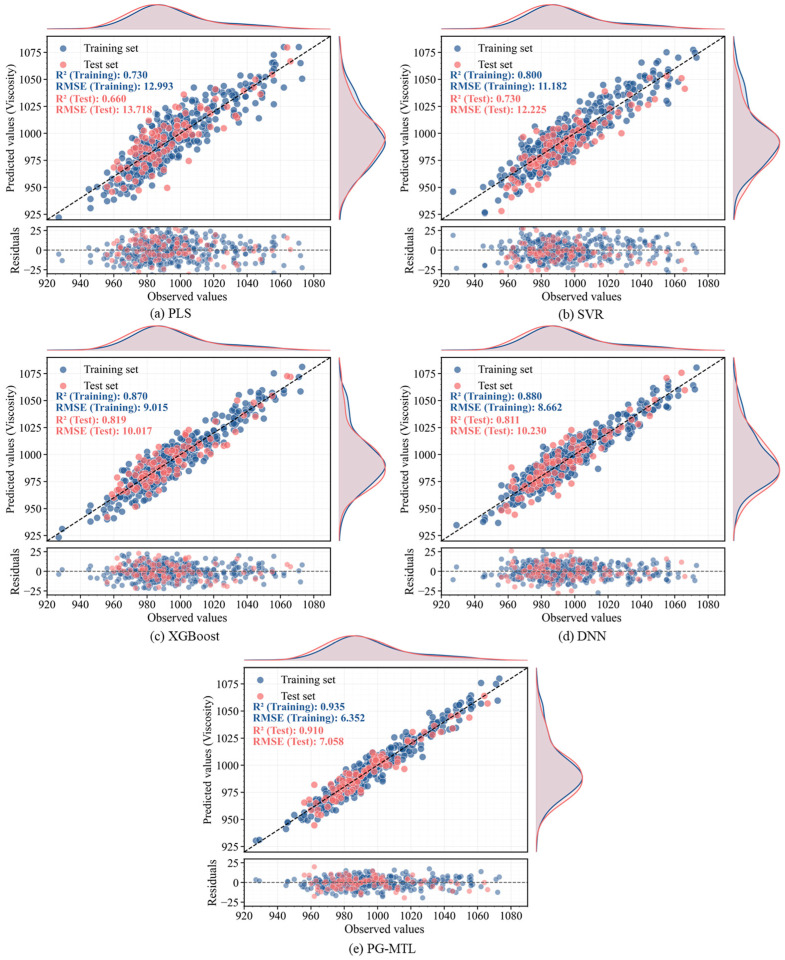
Parity and residual plots for Pulp Viscosity prediction: (**a**) PLS, (**b**) SVR, (**c**) XGBoost, (**d**) MTL-DNN, and (**e**) proposed PG-MTL. Training and test sets are shown in blue and red, respectively.

**Figure 6:**
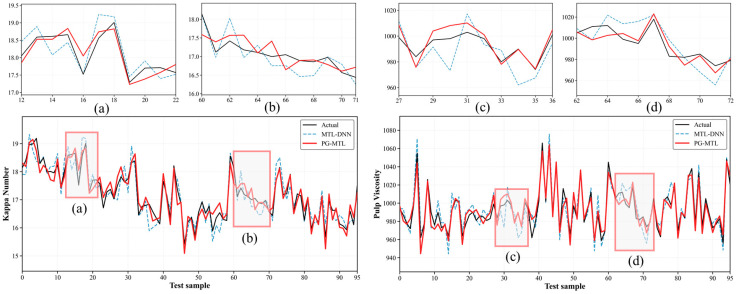
Test-set trajectory tracking results for Kappa number and pulp viscosity. The measured values are compared with the predictions of MTL-DNN and PG-MTL over the full test sequence, and the upper panels provide enlarged views of the representative regions marked as (a–d).

**Figure 7:**
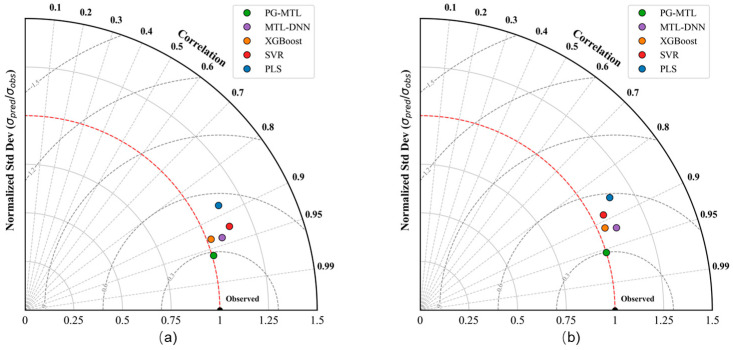
Normalized Taylor diagrams for the test-set predictions of (**a**) Kappa number and (**b**) pulp viscosity. Each point represents one model and summarizes its structural agreement with the observed data in terms of the correlation coefficient, normalized standard deviation, and centered root-mean-square difference. Points closer to the reference point (“Observed”) indicate better overall reproduction of the statistical structure of the measured signals.

The overall predictive performance of the proposed PG-MTL framework was evaluated by comparison with four representative benchmark models, namely PLS, SVR, XGBoost, and MTL-DNN. The quantitative results are summarized in Table 3, and the corresponding visual comparisons are presented in Figure 4, Figure 5, Figure 6 and Figure 7 through parity and residual plots, test-set trajectory tracking curves, and Taylor diagrams.

As shown in Table 3, PG-MTL achieved the best predictive performance for both Kappa number and pulp viscosity. For the Kappa number, the model yielded an RMSE of 0.245, an MAE of 0.204, and an $R^{2}$ of 0.920. The corresponding values for pulp viscosity were 7.058, 5.491, and 0.910. Compared with the strongest benchmark model, PG-MTL reduced the RMSE by 23.2% for the Kappa number and 29.5% for pulp viscosity. Relative to the data-driven MTL-DNN baseline, the RMSE reductions were 24.6% and 31.0%, respectively. Similar improvements were also observed in MAE and $R^{2}$. These results indicate that the advantage of PG-MTL was consistent across both targets and remained evident relative to both conventional benchmark models and the multi-task deep baseline.

The parity and residual plots in Figure 4 and Figure 5 further support the numerical results. For both Kappa number and pulp viscosity, the PG-MTL predictions cluster most closely around the ideal diagonal line in both the training and test sets, suggesting better agreement between measured and predicted values. In contrast, PLS and SVR show noticeably larger scatter, whereas XGBoost and MTL-DNN fall in between. The residual plots also show that the errors of PG-MTL are more tightly centered around zero, without any clear systematic drift across the operating range. This is consistent with the lower RMSE and MAE values reported in Table 3.

To further evaluate predictive performance under realistic operating conditions, Figure 6 shows the test-set trajectory tracking results for the measured values, MTL-DNN predictions, and PG-MTL predictions, together with local enlargements of representative segments. Only MTL-DNN and PG-MTL are compared here. This is because they share the same multi-task backbone and differ mainly in whether physics-guided regularization is introduced, making it possible to assess the effect of the proposed physics-guided design on temporal tracking more directly. For both Kappa number and pulp viscosity, PG-MTL follows the measured trajectories more closely over the entire test sequence. This difference is more evident in the enlarged regions (a) and (b). In these segments, the proposed model captures local turning points, short-term fluctuations, and variation amplitudes more accurately. By comparison, although MTL-DNN reproduces the overall trend reasonably well, it shows larger deviations near abrupt changes and local extrema, especially in the highlighted regions. Overall, the closer agreement between PG-MTL and the measured trajectories suggests that physics-guided regularization, together with joint feature learning, is beneficial for dynamic quality tracking.

To further evaluate predictive performance from a structural perspective, normalized Taylor diagrams were used, as shown in Figure 7, with panel (a) for Kappa number and panel (b) for pulp viscosity. These diagrams summarize three statistics simultaneously: the correlation coefficient *r*, the standard deviation ratio *σ_pred_*/*σ_obs_*, and the normalized centered root-mean-square difference (CRMSD/σ_obs_). On the test set, PG-MTL gave the best overall structural match for Kappa number, with *r* = 0.9603, a standard deviation ratio of 1.0079, and the smallest normalized CRMSD (0.2828). Similar results were obtained for pulp viscosity, where PG-MTL reached *r* = 0.9551 and *σ_pred_*/*σ_obs_* = 1.0001. These values indicate that the model reproduced both the trend and the variability of the measured signal well. MTL-DNN also showed relatively high correlation, but its standard deviation ratio deviated more from unity, especially for viscosity prediction (1.0925), indicating weaker structural consistency. Taken together, the Taylor-diagram results show that PG-MTL achieved the most balanced agreement with the observed data.

The quantitative results in Table 3 and the visual evidence in Figure 4, Figure 5, Figure 6 and Figure 7 support the same overall conclusion. Among the compared models, PG-MTL provides the most accurate and structurally reliable predictions for both Kappa number and pulp viscosity. Its advantage is reflected not only in lower point-wise prediction errors, but also in tighter agreement with the measured trajectories and closer consistency with the statistical characteristics of the observed industrial data. In this sense, the improvement brought by PG-MTL is not limited to numerical accuracy alone. It also enhances dynamic tracking performance and structural fidelity, both of which are important for reliable soft sensing under realistic industrial operating conditions.

### 4.2. Ablation Study on Model Components

To examine the respective contributions of the multi-task architecture and the physics-guided design, an ablation study was conducted by comparing the proposed PG-MTL model with two reduced variants:

- Model A (Single-Task DNN): a standard deep neural network trained only for Kappa number prediction, without auxiliary tasks or physics-guided regularization.
- Model B (Data-Driven MTL): a multi-task model that predicts both Kappa number and pulp viscosity through a shared representation, optimized using only the data-driven loss *L*_*data*_.
- Model C (PG-MTL): the complete proposed model, which combines the multi-task architecture with the physics-guided loss *L_phy_*.

**Figure 8:**
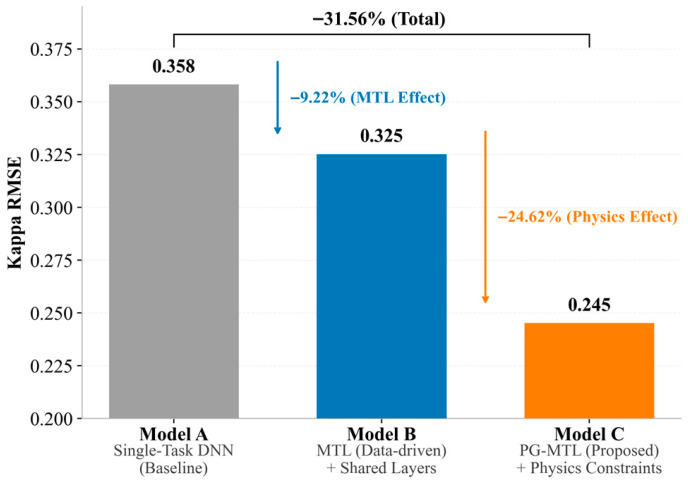
Ablation study showing the stepwise contributions of multi-task learning and physics-guided regularization to Kappa number prediction performance.

The quantitative comparison is summarized in Table 4 and visualized in Figure 8. For Kappa number prediction, the single-task DNN (Model A) yielded a baseline RMSE of 0.358. Introducing viscosity prediction as an auxiliary task reduced the RMSE to 0.325 in Model B, corresponding to a 9.22% improvement over the baseline. This gain suggests that shared representation learning can exploit process information that is relevant to both targets, thereby improving the generalization of the primary Kappa prediction task. When physics-guided regularization was further introduced, the RMSE decreased from 0.325 to 0.245 in Model C, corresponding to an additional 24.62% reduction relative to Model B. A consistent improvement was also observed for pulp viscosity, whose RMSE decreased from 10.230 in Model B to 7.058 in Model C.

Taken together, the ablation results suggest that the overall performance gain arises from two complementary sources. The transition from Model A to Model B reflects the contribution of shared representation learning across the two related targets, whereas the transition from Model B to Model C reflects the additional contribution of physics-guided regularization. In this sense, the multi-task architecture provides a useful representational foundation, while the larger incremental improvement under the present setting is associated with the physics-guided component. These results indicate that the multi-task architecture provides a useful foundation for feature learning, while the physics-guided component supplies a substantial additional gain, especially under limited-data conditions. A similar pattern is observed for pulp viscosity, indicating that the benefit of the proposed framework does not arise from a single target alone, but from the combined effect of task coupling and physics-guided regularization. In other words, shared learning improves representation quality, whereas the physics-guided term further regularizes the model toward kinetically plausible solutions.

### 4.3. Robustness Under Small-Sample Conditions

**Figure 9:**
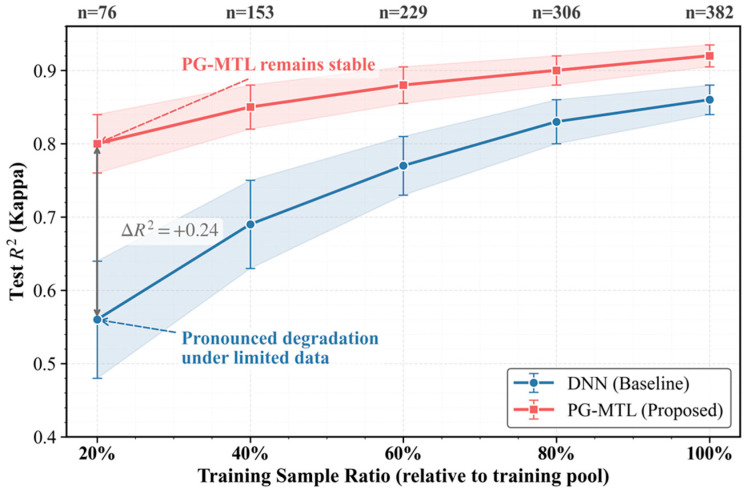
Robustness evaluation under progressively reduced training data. The curves show model performance as a function of the available training-set size, and the shaded regions/error bars indicate ±1 standard deviation over repeated random subsampling trials.

To evaluate the robustness of the proposed framework under limited-data conditions, a systematic data-reduction experiment was conducted by progressively decreasing the size of the training set. The complete dataset (*N* = 478) was first divided into a fixed 20% test set and an 80% training pool (*N_train_* = 382). From this training pool, subsets ranging from 20% (*n* = 96) to 100% (*n* = 382) were randomly sampled for model training. Each sampling ratio was repeated five times with different random seeds, and the shaded regions and error bars in Figure 9 denote ±1 standard deviation.

As shown in Figure 9, both models showed performance degradation as the amount of available training data decreased. Here, the comparison focuses on the baseline single-task DNN and PG-MTL in order to examine whether the proposed framework remains effective when labeled samples are limited. The DNN exhibited a clear decline in predictive accuracy together with increased performance variance, and its mean *R*^2^ dropped to approximately 0.56 at the lowest sampling ratio (20%). By contrast, PG-MTL remained more stable across the full range of sampling ratios. Even when only 20% of the training pool was used, it still maintained an *R*^2^ close to 0.80, with noticeably smaller variance. At this sampling ratio, the corresponding RMSE for the Kappa number was 0.391, indicating that the model retained not only relatively strong explanatory power but also a practically meaningful level of absolute prediction accuracy under severe data reduction.

The resulting performance gap (Δ*R*^2^ ≈ 0.24) suggests that PG-MTL is better suited to small-sample conditions than the baseline DNN. This advantage is likely related to the combined effect of shared representation learning and physics-guided regularization, which provides a more structured learning bias when supervision is sparse. As the amount of training data decreases, the superiority of PG-MTL becomes more evident. This result highlights its practical value for industrial soft-sensing applications, where labeled samples are often costly and inherently limited.

### 4.4. Physical Consistency Verification

To further assess the physical plausibility of the proposed PG-MTL framework, an H-factor stress test was conducted to examine whether the learned mapping preserves a fundamental kinetic trend of kraft cooking. The H-factor summarizes time–temperature severity and is widely used as an integrated indicator of cooking intensity. Under otherwise fixed operating conditions, a higher H-factor is generally associated with stronger delignification and thus with a non-increasing Kappa number. In the present context, this corresponds to the monotonic physical constraint introduced in Equation (6). For the present stress test, this constraint was evaluated in discrete form over the selected H-factor sweep.

**Figure 10:**
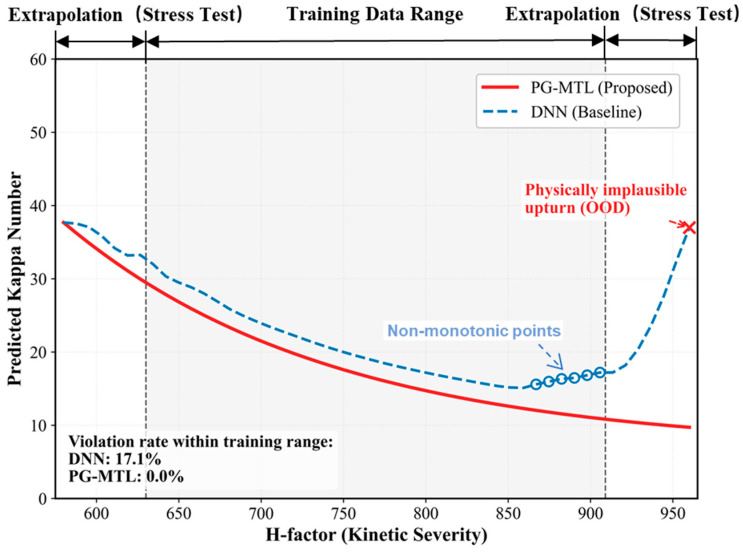
Physical consistency verification via H-factor stress testing and extrapolation beyond the empirical training range. The shaded regions indicate intervals outside the H-factor range covered by the training data.

A representative operating point was selected from the central region of the test-set distribution, while all process variables except the H-factor were held constant. The H-factor was then swept over the interval 580–960, and the predicted Kappa responses were recorded for (i) a purely data-driven single-task DNN baseline and (ii) the proposed PG-MTL model. The empirical H-factor range covered by the training data was 630–909. As indicated in Figure 10, this interval was treated as the training-supported region, whereas the intervals 580–630 and 909–960 were regarded as extrapolative regions. In these extrapolative regions, the analysis focuses on trend plausibility and stability rather than quantitative accuracy.

Within the training-supported interval, PG-MTL produced a smooth and monotonically decreasing Kappa trajectory as the H-factor increased, which is consistent with the expected delignification trend. By contrast, the DNN baseline showed several local non-monotonic segments in the same interval, where the predicted Kappa increased despite an increase in H-factor. To quantify this behavior, a monotonicity violation rate (VR) was defined using the discrete slopes along the H-factor sweep. Let ${\left\{H_{i}\right\}}_{i=1}^{N}$ denote a strictly increasing sequence of H-factor values, and let {${\left\{{\hat{y}}_{k,i}\right\}}_{i=1}^{N}$} denote the corresponding model predictions. The discrete slope between two adjacent points is defined as:

(18) $$s_{i}=\frac{{\hat{y}}_{k,i+1}-{\hat{y}}_{k,i}}{H_{i+1}-H_{i}},i=1,\ldots ,N-1.$$

A physically plausible response requires $s_{i\;}$≤ 0. The violation rate is then defined as:

(19) $$VR=\frac{1}{N-1}\sum_{i=1}^{N-1}I(s_{i}>0),$$

where I(·) denotes the indicator function. Within the training-supported interval, the DNN baseline exhibited a violation rate of 17.1%, whereas PG-MTL achieved 0.0%. This result indicates that the proposed framework better preserves the expected monotonic trend in the data-supported regime.

Beyond the upper boundary of the training-supported interval, the DNN baseline developed an upward tail, with predicted Kappa increasing as the H-factor continued to rise. Although no reference data are available in this region to assess extrapolation accuracy quantitatively, such a trend reversal is physically undesirable because it contradicts the expected cooking kinetics. Under the same stress test, PG-MTL maintained a decreasing tendency, suggesting that the physics-guided design helps stabilize the model response near the edge of the training-supported region.

It should also be noted that this behavior was learned through the training objective itself rather than imposed afterward by manual smoothing. Overall, the H-factor stress test suggests that PG-MTL improves not only predictive accuracy but also local behavioral consistency with known pulping kinetics. This property is particularly relevant for industrial soft-sensing applications operating under variable process conditions.

## 5. Conclusions

This study developed a physics-guided multi-task learning framework (PG-MTL) for the joint soft-sensing prediction of Kappa number and pulp viscosity in continuous kraft pulping. By combining hard-parameter-sharing representation learning with an H-factor-based monotonicity constraint, the framework was intended to address two practical challenges in industrial soft sensing: limited labeled data and insufficient physical consistency in purely data-driven models.

Results from industrial operating data showed that PG-MTL performed best among the compared models. On the independent test set, it achieved an *R*^2^ value of 0.920 for the Kappa number and 0.910 for pulp viscosity. The model also showed better temporal tracking and closer structural agreement with the measured signals. Ablation and reduced-sample analyses further indicated that both the multi-task design and the physics-guided constraint contributed to the final performance, with the latter becoming more important when training data were scarce. Even when only 20% of the training data were retained, PG-MTL still maintained an *R*^2^ close to 0.80. The H-factor stress test further showed that the monotonicity violation rate decreased from 17.1% in the unconstrained baseline to 0.0% within the evaluated range.

These findings suggest that introducing process knowledge into multi-task learning is beneficial not only for prediction accuracy, but also for physical plausibility and robustness. Under small-sample industrial conditions, the proposed PG-MTL framework offers a practical solution for dual-quality prediction in continuous kraft pulping.

The present study was carried out within a relatively specific industrial setting, and several aspects remain to be explored further. The model was developed and validated using data from a single production line, so its transferability across different digesters, wood species, and operating regimes still needs systematic verification. In addition, the current physics-guided design is centered mainly on the monotonic relationship between H-factor and Kappa number, whereas the coupling term between Kappa number and pulp viscosity remains partly empirical. The physical-consistency analysis was also evaluated within the supported operating range and its immediate extrapolative neighborhood, and therefore should not be interpreted as a general guarantee of prediction reliability under all off-design conditions. More broadly, extending the proposed framework to other industrial processes would require identifiable process-specific priors, sufficiently informative process variables, and validation under the corresponding operating regimes. In this respect, uncertainty quantification would be particularly valuable for indicating model confidence when the process deviates from the training-supported region or operates under dynamically changing conditions.

Future work will extend the framework in directions that are directly relevant to industrial applications. One important step is to incorporate additional physicochemical constraints so that viscosity evolution and other quality-related variables can be described more explicitly. Another is to test the transferability of the proposed framework across different operating lines, wood species, and digester configurations. It would also be of practical interest to integrate the proposed soft sensor into a closed-loop model predictive control framework for real-time cooking optimization. Improved prediction accuracy may support tighter operation around desired quality targets, although no preliminary closed-loop simulation or economic assessment was conducted in the present study. The corresponding control-level and economic benefits therefore remain to be validated separately in future work. Further work will also consider the incorporation of prediction confidence intervals or uncertainty estimates to improve the practical interpretability and deployment reliability of the proposed soft sensor.

## Figures and Tables

**Table 1:** Summary of selected input and output variables for the soft sensor model.

Symbol	Variable Description	Role	Unit	Physical Significance	MIN	MAX
$x_{H}$	H-factor	Input	-	Integrated thermal kinetic driver	630.00	909.00
$x_{EA}$	Effective Alkali	Input	g/L	Initial chemical driving force	105.51	111.98
$x_{S}$	Sulfidity	Input	%	Catalyst for delignification & carbohydrate protector	33.92	37.13
$x_{RA,1}$	Residual Alkali-Impregnation Top	Input	g/L	Early-stage consumption feedback	8.73	17.57
$x_{RA,2}$	Residual Alkali-Impregnation Bottom	Input	g/L	Impregnation completion indicator	5.44	10.41
$x_{RA,3}$	Residual Alkali-Upper Cook	Input	g/L	Cooking zone concentration profile	10.52	13.62
$x_{RA,4}$	Residual Alkali-Lower Cook	Input	g/L	Bulk delignification status	5.99	8.97
$x_{RA,5}$	Residual Alkali-Extraction	Input	g/L	Final reaction state proxy	4.56	5.59
$y_{k}$	Kappa Number	Output	-	Lignin content indicator	15.05	18.64
$y_{v}$	Pulp Viscosity	Output	mL/g	Cellulose degree of polymerization	945.00	1072.00

**Table 2:** Hyperparameter values used in the developed models.

Algorithm	Hyperparameter	Value/Search Space
PG-MTL Network	Hidden layers (Shared trunk)	64–32
	Hidden layer (Task heads)	16
	Activation function	Tanh
	Initial learning rate	0.001
	Optimizer	Adam
	Learning rate scheduler:	ReduceLROnPlateau (factor = 0.5, patience = 10)
	Batch size	32
	Maximum epochs/Patience	500/30 epochs
	Weight decay (L2)	1 × 10^−4^
Physics Constraints	Monotonicity penalty weight ($\lambda_{1})$	0.1
	Correlation penalty weight ($\lambda_{2}$)	0.05
	Minimum correlation bound ($\rho_{min}$)	0.35
MTL-DNN	Network Architecture	Identical to PG-MTL
	Physics penalties ($\lambda_{1},\lambda_{2}$)	0
XGBoost	Number of estimators (n_estimators)	[50, 100, 200, 500]
	Maximum depth (max_depth)	[3, 5, 7, 9]
	Learning rate (learning_rate)	[0.01, 0.1, 0.2]
	Subsample	[0.8, 1.0]
SVR (RBF Kernel)	Regularization parameter (C)	[0.1, 1, 10, 100]
	Kernel coefficient (γ)	[‘scale’, ‘auto’, 0.01, 0.1, 1]
PLS	Number of latent components	[3, 4, 5, 6, 7]

**Table 3:** Predictive performance comparison of the proposed PG-MTL framework and benchmark models on the independent test set.

Model	Type	Kappa Number ($y_{k}$)			Pulp Viscosity ($y_{v}$)		
		RMSE	MAE	$R^{2}$	RMSE	MAE	$R^{2}$
PLS	Linear	0.467	0.392	0.710	13.718	10.904	0.660
SVR	Kernel	0.378	0.313	0.810	12.225	9.539	0.730
XGBoost	Ensemble	0.319	0.264	0.865	10.017	8.121	0.819
MTL-DNN	Deep Learning	0.325	0.270	0.859	10.230	8.245	0.811
PG-MTL	Proposed	0.245	0.204	0.920	7.058	5.491	0.910

**Table 4:** Performance comparison of the ablated models demonstrating the effectiveness of the proposed PG-MTL components.

Model	Architecture	Components	Loss Function	Kappa RMSE	RMSE Reduction (%)	Viscosity RMSE	RMSE Reduction (%)
Model A	Single-Task DNN(Kappa)	Baseline	*L_data_*	0.358	-	N/A	-
Model B	MTL-DNN (Data-driven)	+Shared Layers	*L_data_*	0.325	9.22%	10.230	Baseline
Model C	PG-MTL	+Physics-guided regularization	*L_total_*	0.245	31.56% (Total)	7.058	31.01%

## Data Availability

The data presented in this study are available on request from the corresponding author. The data are not publicly available due to privacy.
